# Cardiovascular Risk Associated with Alpha-1 Antitrypsin Deficiency (AATD) Genotypes: A Meta-Analysis with Meta-Regressions

**DOI:** 10.3390/jcm12206490

**Published:** 2023-10-12

**Authors:** Pasquale Ambrosino, Giuseppina Marcuccio, Carmen Lombardi, Silvestro Ennio D’Anna, Stefano Sanduzzi Zamparelli, Costantino Mancusi, Giorgio Alfredo Spedicato, Andrea Motta, Mauro Maniscalco

**Affiliations:** 1 Istituti Clinici Scientifici Maugeri IRCCS, Directorate of Telese Terme Institute, 82037 Telese Terme, Italy; 2 Istituti Clinici Scientifici Maugeri IRCCS, Pulmonary Rehabilitation Unit of Telese Terme Institute, 82037 Telese Terme, Italy; giuseppina.marcuccio@icsmaugeri.it (G.M.); carmen.lombardi@icsmaugeri.it (C.L.); silvestro.danna@icsmaugeri.it (S.E.D.); 3 Department of Clinical Medicine and Surgery, Federico II University, 80131 Naples, Italy; stefanosanduzzi@gmail.com; 4 Department of Advanced Biomedical Science, Federico II University, 80131 Naples, Italy; costantino.mancusi@unina.it; 5 Department of Statistics and Quantitative Methods, Milano-Bicocca University, 20126 Milan, Italy; spedicato_giorgio@yahoo.it; 6 Institute of Biomolecular Chemistry, National Research Council, 80078 Pozzuoli, Italy; andrea.motta@icb.cnr.it

**Keywords:** alpha-1 antitrypsin deficiency, chronic obstructive pulmonary disease, chronic disease, cardiovascular disease, ischemic heart disease, disability, rehabilitation, exercise, outcome

## Abstract

Background. Alpha-1 antitrypsin deficiency (AATD) can result in severe liver and respiratory disorders. The uninhibited elastase activity on the elastic tissue of arterial walls suggests that AATD may also impact vascular health. Thus, we performed a meta-analysis of the studies evaluating cardiovascular risk in individuals with AATD and non-AATD controls. Methods. A systematic literature search was conducted in the main scientific databases according to the Preferred Reporting Items for Systematic Reviews and Meta-Analyses (PRISMA) guidelines. Differences between cases and controls were expressed as odds ratios (OR) with 95% confidence intervals (95%CI). The protocol was registered on PROSPERO under the identification number CRD42023429756. Results. The analysis of eight studies showed that, with a prevented fraction of disease of 15.0% and a corresponding OR of 0.779 (95%CI: 0.665–0.912; *p* = 0.002), a total of 24,428 individuals with AATD exhibited a significantly lower risk of ischemic heart disease compared to 534,654 non-AATD controls. Accordingly, given a prevented fraction of disease of 19.5%, a lower risk of acute myocardial infarction was documented when analyzing four studies on 21,741 cases and 513,733 controls (OR: 0.774; 95%CI: 0.599–0.999; *p* = 0.049). Sensitivity and subgroup analyses substantially confirmed results. Meta-regression models suggested that these findings were not influenced by AATD genotypes or prevalence of chronic obstructive pulmonary disease (COPD) among cases and controls, while higher differences in the prevalence of male sex (Z-score: 3.40; *p* < 0.001), hypertension (Z-score: 2.31; *p* = 0.021), and diabetes (Z-score: 4.25; *p* < 0.001) were associated with a lower effect size. Conclusions. Individuals with AATD may exhibit a reduced risk of ischemic heart disease, even in the presence of mild deficiency of the serine protease inhibitor. Although caution is warranted due to the observational nature of the data, future pharmacological and rehabilitation strategies should also take this controversial relationship into account.

## 1. Introduction

Alpha-1 antitrypsin deficiency (AATD) is a genetic disorder that exhibits an autosomal codominant inheritance pattern, causing reduced levels of alpha-1 antitrypsin (AAT) in the bloodstream [1]. AAT is the most abundant serine protease inhibitor found in the blood and is encoded by the Serpin Family A Member 1 (SERPINA1) gene located on chromosome 14 [2]. Based on electrophoretic protein band positions, the most commonly observed genetic variants of AAT are the wild-type alleles M and the single nucleotide polymorphisms Z (Glu342Lys) and S (Glu264Val) [3,4]. With an estimated prevalence ranging from less than 0.1% to 0.25% in Europe [5], the PiZZ genotype is a rare but highly detrimental condition associated with a significant decrease in serum AAT concentration [6]. Therefore, although over 150 mutations have been so far identified, this genotype defines the most common severe form of AATD [7]. Conversely, genotypes such as PiSZ, PiMZ, and PiSS lead to an intermediate or mild deficiency, with potential clinical relevance but lower severity [8].

Regardless of severity, the fundamental pathological mechanism in individuals with AATD is an imbalance between proteases and antiproteases [1], along with the absence of a crucial anti-inflammatory factor during acute-phase responses and the retention of misfolded AAT protein [9]. These alterations may give rise to various clinical manifestations, especially early onset chronic obstructive pulmonary disease (COPD) and liver cirrhosis [10,11,12]. Nevertheless, epidemiological evidence suggests that individuals with AATD may have several additional clinical manifestations, such as vasculitis and cancer [13], especially those with more severe forms of the disease [14].

Given the uninhibited elastase activity on the elastic tissue of the arterial wall [15], AATD may also impact the elastic properties of larger blood vessels, thus somehow influencing vascular health and the cardiovascular disease burden in this clinical setting [16]. In particular, it has been hypothesized that, when the proportion of elastin and collagen in the blood vessels is altered, their distensibility is affected, potentially leading to a decrease in blood pressure [17]. This condition, which offers protection against cardiovascular events, may indicate a reduced ischemic risk in individuals with AATD [18]. However, since the link between AATD and low blood pressure has been disputed in other research [19], alternative theories have also emerged to account for the reduced cardiovascular disease burden documented in large nationwide cohorts [17,18]. Among these, one should contemplate the compromised hepatic lipid secretion in AATD, resulting in lower levels of triglycerides and low-density lipoprotein cholesterol in the bloodstream, or the heightened medical supervision of individuals with severe disease [18,20]. In this scenario, it should be considered that AAT also plays an unquestionable cytoprotective role in the vascular endothelium [21], mitigating the harm caused by ischemia and reperfusion during myocardial infarction [22] and counteracting the progression of coronary disease [23]. Furthermore, it is important to note that COPD, which is the most frequent clinical manifestation of AATD, acts by itself as an independent risk factor for cardiovascular disease [24,25]. Thus, both prospective [18,26,27,28] and retrospective studies [17,29,30,31] attempted to analyze the impact of AATD on subclinical atherosclerosis and cardiovascular risk, in consideration of the potential vascular repercussions of AATD and the conflicting epidemiological evidence on this matter [19,23].

Given the information provided, we planned to conduct a systematic review with meta-analysis of all studies evaluating the prevalence of ischemic heart disease in individuals with AATD and non-AATD controls. Furthermore, we intended to include meta-regression analyses to examine the influence of various genotypes, as well as important clinical and demographic variables, on the observed results.

## 2. Materials and Methods

We created a protocol with defined objectives and inclusion criteria to conduct a detailed literature research. The protocol predetermined the outcomes, statistical analyses, and methods for evaluating study quality, and it was registered on PROSPERO under the identification number CRD42023429756.

### 2.1. Search Strategy

Following the Preferred Reporting Items for Systematic Reviews and Meta-Analyses (PRISMA) guidelines, we conducted a comprehensive literature search in the primary scientific databases, including PubMed, Web of Science, Scopus, and EMBASE [32]. We used the following search terms in any possible combination: α1 antitrypsin deficiency, α1-antitrypsin deficiency, alpha-1 antitrypsin deficiency, alpha-1-antitrypsin deficiency, myocardial, infarction, angina, cardiac, cardiovascular, ischemic, and ischemia. To ensure that no articles were inadvertently excluded, we opted not to apply any language restrictions during the search. The last search was conducted on 4 October 2023 (Table S1). In addition, we conducted a review of the reference lists of the chosen articles. For studies that showed potential for inclusion in the meta-analysis, we contacted the authors to acquire any missing data. The article texts were evaluated by two independent investigators (GM and SSZ), who extracted the data separately. In case of discrepancy between the two investigators, a third one (CL) was consulted. Overall, we achieved a high inter-reader agreement (κ = 0.97). The outcomes of the literature search were reported in accordance with the PRISMA flowchart.

### 2.2. Extraction of Data and Quality Assessment

Following a pre-specified protocol, all studies evaluating the prevalence of ischemic heart disease in individuals with AATD and non-AATD controls were considered. Abstracts and posters from scientific conferences were also evaluated, while case series without a control group, case reports, animal studies, and reviews were excluded. The main outcome measure was defined as the presence of any acute, subacute, and/or chronic heart disease of ischemic etiology, including stable or unstable angina pectoris, coronary atherosclerosis, coronary thrombosis, and acute myocardial infarction with its concurrent or subsequent complications (e.g., hemopericardium, heart aneurysm or dissection, rupture of chordae tendineae, or rupture of papillary muscle). Where possible, data on sample size and AATD genotype, as well as key demographic and clinical variables related to comorbidities and lung function, were extracted in all included studies.

To assess the methodological quality of the non-randomized observational studies, we employed the Newcastle–Ottawa Scale (NOS). This scale provided a standardized framework for evaluating the quality of the articles [33]. In summary, the scoring system of the NOS assessed three main areas: selection (4 records), comparability (1 record), and exposure (3 records) for case–control studies or outcome (3 records) for cohort ones. Each individual record within these areas could receive a maximum score of 1, except for comparability, which allowed a maximum of 2 points. The final scores were obtained by summing up the scores of each record, resulting in a total score ranging from 0 (indicating the lowest possible quality) to 9 (indicating the highest possible quality).

### 2.3. Meta-Analysis and Publication Bias

Statistical analyses were carried out with Comprehensive Meta-analysis Version 3 (Biostat Inc., Englewood, NJ, USA) and Review Manager Version 5.4.1 (The Cochrane Collaboration, Copenhagen, Denmark). In each group, the absolute risk in cases and controls was calculated as [(number of events)/(total number of subjects)] × 100. The attributable risk, namely the percentage of disease in the exposed group that can be attributed to the exposure, was defined as [(number of events in cases—number of events in controls)/(number of events in cases)] × 100. In case of a negative attributable risk, the prevented fraction of disease was calculated as [(incidence rate in controls—incidence rate in cases)/(incidence rate in controls)] × 100, thus expressing the proportion of cases of the disease that have been prevented by the exposure [34]. To analyze the differences between cases and controls, we utilized odds ratios (OR) along with their corresponding 95% confidence intervals (95%CI). These measures allowed us to estimate the strength and direction of the association between variables. Additionally, we calculated the prediction intervals, which provide a range indicating where the results of future studies are likely to fall [35]. To ensure a conservative approach, we employed the random effects method, which takes into account both within-study and between-study variance [36].

The overall effect was assessed using Z-scores, with statistical significance set at a *p*-value ≤ 0.05. In order to account for statistical heterogeneity among the studies, we conducted the chi-square Cochran’s Q test and calculated the I^2^ index. The I^2^ index quantifies the inconsistency among the study results and represents the proportion of total variation in estimates that can be attributed to heterogeneity rather than sampling error. A brief interpretation of the I^2^ values is as follows: 0% indicates the absence of heterogeneity, values below 25% suggest low heterogeneity, values from 25% to 50% indicate moderate heterogeneity, and values greater than 50% indicate high heterogeneity [37].

To investigate potential sources of heterogeneity, we performed additional analyses by excluding one study at a time. We also visually examined funnel plots, which display the relationship between effect size and precision (1/standard error of the log odds ratio), to identify any asymmetry and assess for potential small-study effects. Furthermore, we conducted the Egger’s regression test and the Begg and Mazumdar rank correlation test to quantitatively evaluate publication bias, in addition to subjective assessment. A *p*-value <0.10 was considered statistically significant for these tests [38]. Finally, we turned to the Duval and Tweedie’s trim-and-fill analysis to calculate an adjusted effect size after trimming and imputing unpublished studies [39].

### 2.4. Sensitivity Analyses

In order to investigate potential sources of heterogeneity, we planned to repeat the analyses by including only “better quality” studies according to NOS (i.e., NOS ≥ median value found among included studies). Additionally, another sensitivity analysis was performed after excluding the studies enrolling individuals with AATD primarily or exclusively on the basis of serum AAT levels or medical history. To ensure no data overlap, we also decided to conduct an additional analysis by excluding the studies where the participants could potentially be a subset of those enrolled in other studies conducted within the same region or country, but with a larger sample size.

### 2.5. Subgroup Analyses

Finally, we planned to separately analyze the included studies according to their prospective or retrospective design.

### 2.6. Meta-Regression Analyses

We also speculated that our results on ischemic risk in AATD may be influenced by the differences between cases and controls in major demographic (male gender, mean age) and clinical variables related to cardiovascular risk (hypertension, dyslipidemia, diabetes, smoking, and body mass index (BMI)), pulmonary function (forced expiratory volume in 1 s (FEV_1_), forced vital capacity (FVC), and FEV_1_/FVC), and exercise capacity (6 min walking distance (6MWD)). Similarly, we considered the possibility that the difference between cases and controls may be affected by disease severity and, therefore, by specific AATD genotypes (i.e., PiZZ, PiSZ, PiMZ, and PiSS) and/or augmentation therapies. Finally, we explored the hypothesis that the rate of patients diagnosed with COPD among cases and controls could somehow influence our results. In order to test the possible effect of the above variables in explaining the results observed across studies, we planned to perform meta-regression analyses after implementing regression models with the differences in the prevalence of cardiovascular disease as dependent variables (y) and the above covariates as independent ones (x). For multivariable meta-regressions, we planned to evaluate multicollinearity using the variance inflation factor (VIF). VIF values exceeding 2.5 would indicate the presence of collinearity that could potentially compromise the reliability of the regression model [40].

## 3. Results

After removing duplicate results, we initially had a total of 881 articles. Among these, 634 studies were excluded as they were deemed irrelevant based on a preliminary assessment of the title and/or abstract. Additionally, 232 articles were excluded as they consisted of comments, case reports, reviews, or studies that did not contain the relevant data of interest. Following a meticulous evaluation of the full texts, an additional 7 studies were deemed unsuitable and subsequently discarded from the analysis.

**Figure 1:**
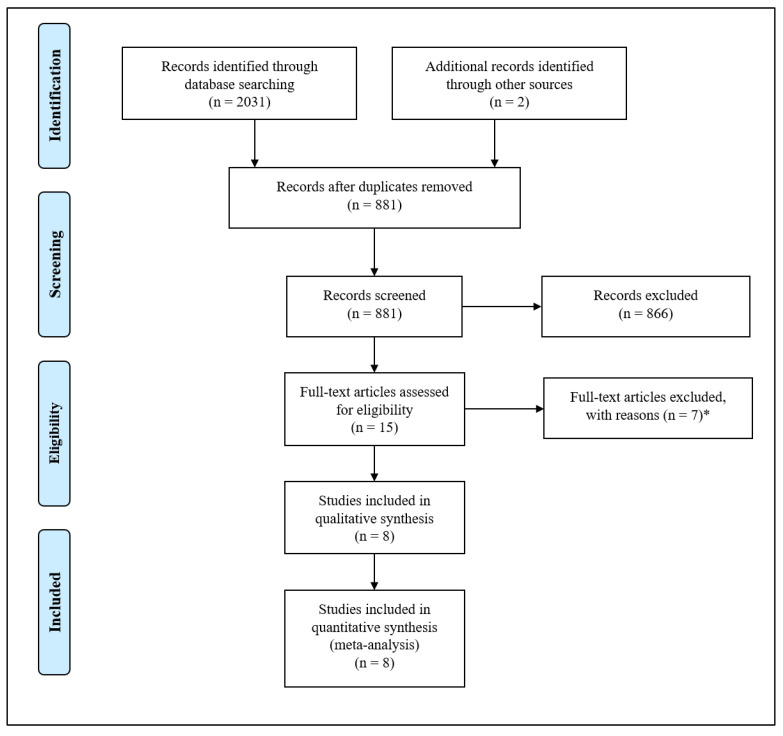
Preferred Reporting Items for Systematic Reviews and Meta-Analyses (PRISMA) flow diagram. * 3 studies without the possibility to retrieve data on the outcomes of interest, 1 study without control group, 1 study on aortic pulse-wave velocity, 1 study on carotid artery intima-media thickness, 1 study on coronary artery ecstasy.

As a result, a total of eight articles [17,18,26,27,28,29,30,31] contained data on ischemic heart disease on a total of 24,428 individuals with AATD and 534,654 non-AATD controls, thus being considered for the final analysis (Figure 1).

### 3.1. Study Characteristics

Among eight included articles, four were prospective [18,26,27,28] while four had a retrospective design [17,29,30,31]. The major demographic and clinical characteristics of the study populations have been reported in Table 1. In the retrieved datasets, the number of individuals with AATD ranged from 6 to 19,003, with a mean age varying between 44.8 and 74.0 years, mean BMI between 24.6 and 27.3 kg/m^2^, and male gender prevalence between 44.1 and 100%. Diabetes was documented in 0–27.3% of participants and hypertension in 4.1–66.7%. The prevalence of COPD ranged between 4.1 and 100%, with 8.4–11.0% current smokers and 34.8–46.1% former smokers.

A complete pulmonary functional assessment was performed in only two studies [27,31], with spirometry parameters and measures of exercise capacity being reported in Table S2. As shown in Table 2, data on AATD genotype were available in all but one [30] article, with two studies [27,28] identifying AATD primarily or exclusively on the basis of serum AAT levels or medical history. Collectively, the PiZZ genotype was reported in 0–100% of AATD cases, the PiSZ in 0–52.8%, the PiMZ in 0–100%, and the PiSS in 0–5.3%. In detail, one study [26] specifically and exclusively enrolled PiZZ patients, while in three studies [18,27,28] the PiZZ and PiSZ genotypes accounted for the largest or global proportion of individuals with AATD. Three studies [17,29,31] enrolled predominantly participants with the PiMZ genotype while in another study [27], on the contrary, participants with the latter genotype were included in the COPD control population, of which, however, they represented only a very small percentage (0.3%).

Data on augmentation therapy with AAT were not mentioned in only one study [30]. While most articles specifically reported the absence of any augmentation therapy, one [27] reported its application in a large subset (79.1%) of AATD patients.

As shown in Table S3, ischemic heart disease was reported as a composite outcome in most included studies, namely as the presence of any acute, subacute, and/or chronic heart disease of ischemic etiology, including stable or unstable angina pectoris, coronary atherosclerosis, coronary thrombosis, and acute myocardial infarction with its complications. The International Classification of Diseases (ICD) was followed in five cases [17,18,26,28,30]. One of the studies [30] exclusively reported on chronic ischemic disease and one [28] on acute myocardial infarction, whereas the exact nature of the cardiovascular outcome was not specified in two studies [27,29]. Out of the remaining articles, three of them [18,27,31] additionally presented separate data on acute myocardial infarction, enabling us to analyze distinct datasets specifically for this outcome.

The NOS for quality assessment of included studies had a median value of 6 (Table S4).

### 3.2. Meta-Analysis and Publication Bias

**Figure 2:**
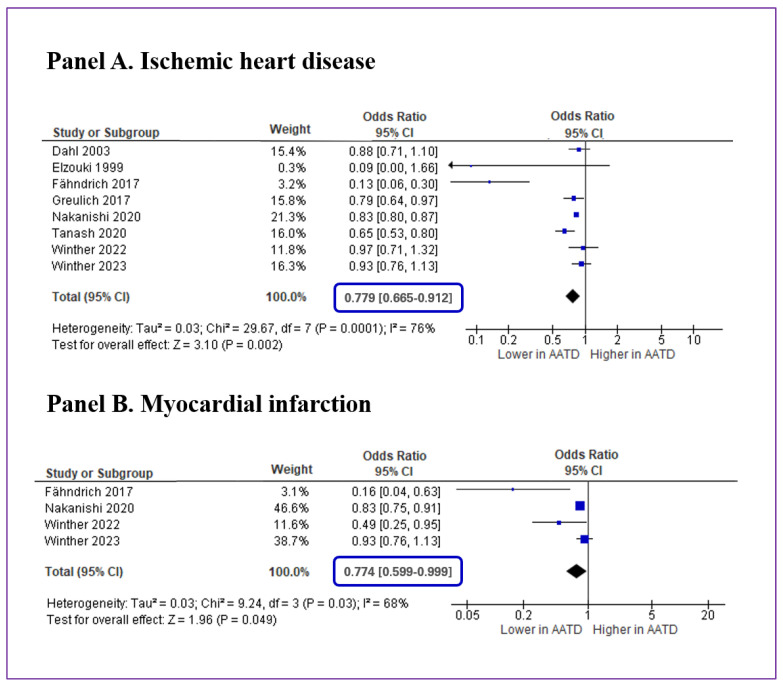
Forest plot of the risk of ischemic heart disease (Panel A) and acute myocardial infarction (Panel B) in individuals with alpha-1 antitrypsin deficiency (AATD) compared with non-AATD controls [17,18,26,27,28,29,30,31]. OR: odds ratio; 95%CI: 95% confidence interval. Each study is graphically represented by a blue square (odds ratio) and a black line (95% confidence interval).

The analysis of eight studies [17,18,26,27,28,29,30,31] showed that the absolute risk of ischemic heart disease was 13.3% (95%CI: 12.9–13.7) in 24,428 individuals with AATD and 15.6% (95%CI: 15.5–15.7) in 534,654 non-AATD controls, with a prevented fraction of disease of 15.0% and a corresponding OR of 0.779 (95%CI: 0.665–0.912; *p* = 0.002, Figure 2A).

**Figure 3:**
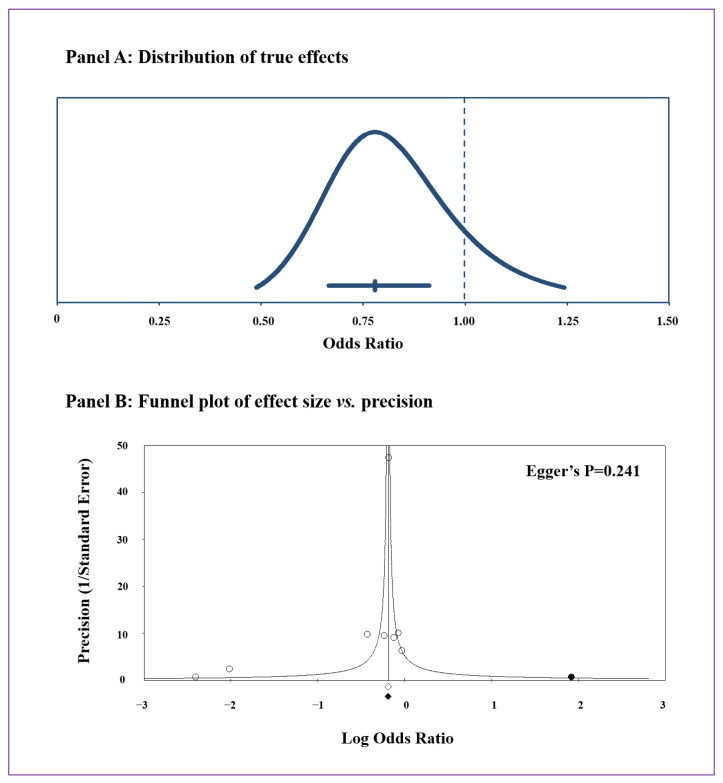
Risk of ischemic heart disease in individuals with alpha-1 antitrypsin deficiency (AATD) compared with non-AATD controls: distribution of true effects in 95% of future comparable populations (Panel A) and funnel plot of effect size vs. precision (1/standard error of the log odds ratio) (Panel B). In Panel B, observed studies and effect size are represented by empty circles and an empty square, respectively. Imputed studies and adjusted effect size are represented by black circles and a black square. respectively.

Computing the prediction interval, we calculated that the true effect size in 95% of future comparable populations would fall between 0.49 and 1.24, thus largely on the left side of the null effect line (Figure 3A). The heterogeneity among the studies was generally high (I^2^: 76.3%, *p* < 0.001). However, after excluding the study by Fähndrich et al. [27], all results were substantially confirmed but heterogeneity was consistently reduced being no longer significant (I^2^: 43.6%, *p* = 0.100). Conducting additional analyses by excluding one study at a time did not yield relevant changes in the overall results on ischemic heart disease.

Given the potential influence of publication bias on meta-analyses results, we visually inspected the funnel plot of effect size vs. precision (1/standard error of the log odds ratio) for studies evaluating ischemic heart disease in order to identify asymmetry (Figure 3B). Being rather symmetrical, we excluded the presence of any publication bias and small-study effect, confirmed by the Egger’s regression test (*p* = 0.241) and the Begg and Mazumdar rank correlation test (*p* = 0.386). Accordingly, the Duval and Tweedie’s trim and fill analysis showed that, after adding one imputed unpublished study, all results were substantially verified (Table S5).

The analysis of four studies [18,27,28,31] reporting separate data on acute myocardial infarction showed that the absolute risk for this outcome was 2.7% (95%CI: 2.5–2.9) in 21,741 cases and 3.3% (95%CI: 3.2–3.4) in 513,733 controls, with a prevented fraction of disease of 19.5% and a corresponding OR of 0.774 (95%CI: 0.599–0.999; *p* = 0.049, Figure 2B). For this additional analysis, we observed substantial heterogeneity (I^2^: 67.3%, *p* = 0.027), while the Egger’s test did not show statistical significance (*p* = 0.248). No other tests for assessing publication bias were conducted due to the limited number of datasets available for this outcome.

### 3.3. Sensitivity Analyses

Given the median value of the NOS quality assessment of 6 (Table S4), we repeated the analyses by including only the studies with a score ≥ 6 [17,18,26,28,30,31]. Interestingly, when separately considering these “better quality” studies, our results on ischemic heart disease were substantially confirmed (OR: 0.826; 95%CI: 0.756–0.903; *p* < 0.001, Table 3A), without significant heterogeneity (I^2^: 40.6%, *p* = 0.135). Similar results with no significant heterogeneity were also obtained after excluding two studies [27,28] identifying AATD primarily or exclusively on the basis of serum AAT levels or medical history (Table 3B). The study by Fähndrich et al. [27], which potentially reported on the same population as another included study with a larger sample [30], was also excluded in the context of an additional analysis aimed at avoiding any data overlap. The exclusion of this study substantially confirmed our results on ischemic heart disease and, interestingly, without significant heterogeneity (Table 3C). Given the limited number of studies reporting separate data on acute myocardial infarction, no sensitivity analysis could be performed for this outcome.

### 3.4. Subgroup Analyses

Considering the differences in study design, we planned to separately analyze prospective and retrospective studies. Interestingly, in the subgroup analysis of prospective studies [18,26,27,28], an even lower risk of ischemic heart disease was reported in 4483 individuals with AATD compared to 36,158 controls (OR: 0.641; 95%CI: 0.421, 0.977; *p* = 0.039, Table 3D). The separate analysis of retrospective studies [17,29,30,31] also confirmed results in 20,192 AATD subjects and 415,120 controls (OR: 0.831; 95%CI: 0.799, 0.865; *p* < 0.001, Table 3E), without heterogeneity (I^2^ = 0%; *p* = 0.430). Due to the limited number of datasets, no subgroup analysis was planned for myocardial infarction.

### 3.5. Meta-Regression Analyses

**Figure 4:**
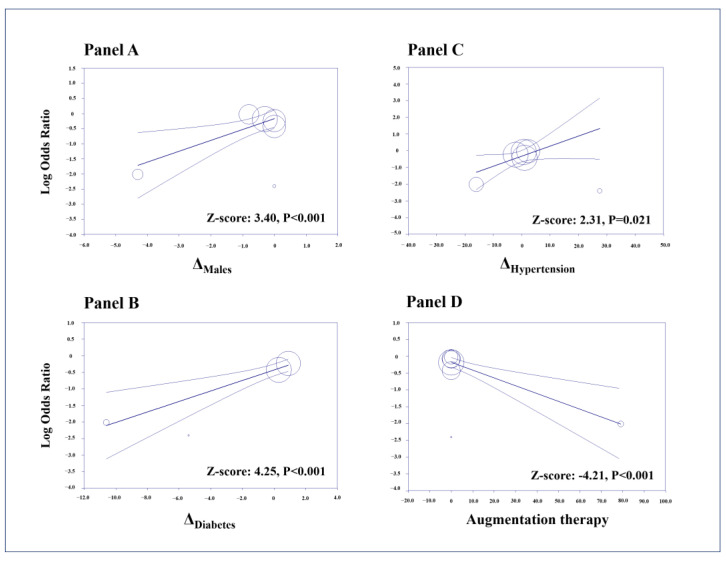
Meta-regression analyses. Impact of differences (Δ) in the prevalence of males (**Panel A**), diabetes (**Panel B**), and hypertension (**Panel C**), and of the prevalence of augmentation therapy in AATD cases (**Panel D**), on the risk of ischemic heart disease in individuals with alpha-1 antitrypsin deficiency (AATD) compared with non-AATD controls.

Meta-regression analyses showed that the findings of our study on ischemic heart disease remained unaffected by any of the considered AATD genotypes (i.e., PiZZ, PiSZ, PiMZ, and PiSS) nor by the disparity in COPD prevalence between individuals with AATD and the control group. On the other hand, higher differences in the prevalence of male sex (Z-score: 3.40; *p* < 0.001, Figure 4A), diabetes (Z-score: 4.25; *p* < 0.001, Figure 4B), and hypertension (Z-score: 2.31; *p* = 0.021, Figure 4C) between cases and controls were associated with a lower effect size. Conversely, a higher rate of patients requiring augmentation therapy determined a larger effect size (Z-score: −4.21; *p* < 0.001, Figure 4D). None of the other tested variables influenced our findings in univariate meta-regressions (Table S6).

In multivariable regression models, assuming that the difference between cases and controls in COPD prevalence was held constant, we found that the differences in the prevalence of males (Z-score: 3.43; *p* < 0.001) and hypertension (Z-score: 3.80; *p* < 0.001) were confirmed as independent predictors of the effect size, with multicollinearity being substantially excluded as a high VIF (i.e., ≥2.5) was not detected. No other multiple regression model could be implemented due to the lack of complete information on all predictors in included studies and to the limited number of datasets. For the same reason, no univariate or multivariable regression model was implemented for myocardial infarction.

## 4. Discussion

Supported by a number of sensitivity and subgroup analyses, this meta-analysis indicates that AATD may have an impact on atherosclerosis progression and global vascular health, with a reduced risk of ischemic heart disease and acute myocardial infarction. Additionally, our meta-regression models suggest that these findings are not influenced by different AATD genotypes or COPD prevalence. On the other hand, it is important to highlight that male sex, hypertension, and diabetes, all recognized as cardiovascular risk factors, tend to diminish such a beneficial cardiovascular effect in this clinical setting. Collectively, these findings support the hypothesis that individuals with AATD, especially those without a low concurrent metabolic risk, could exhibit a decreased burden of cardiovascular disease, even in the presence of only mild deficiency of the serine protease inhibitor.

Our findings align with a clinical and scientific context that has been increasingly focusing on the multitude of manifestations of AATD [41], also considering that this pathological condition, although rare by definition, remains often undiagnosed [42,43]. Pulmonary manifestations are certainly the most frequent, and even individuals with moderate or mild deficiency, such as carriers of the PiSZ or PiMZ genotype, appear to have a significantly increased risk of receiving an early diagnosis of COPD with the classical panacinar emphysema pattern [41]. Another relatively common clinical manifestation is associated with certain variants of the allele, which cause a conformational change in the AAT molecule, leading to its polymerization and retention within hepatocytes [44,45]. The hepatic accumulation of abnormal AAT molecules can cause cholestatic jaundice in the neonatal period, cirrhosis, and hepatocellular carcinoma [44]. In addition to these respiratory and hepatic manifestations, there are many other systemic complications as well, that include but are not limited to cytoplasmic anti-neutrophil cytoplasmic antibody (cANCA)-positive vasculitis, panniculitis, osteoporosis, depression, diabetes, and cancer [13,31]. On the other hand, the relatively low prevalence of AATD in the general population along with the conflicting findings from various literature sources have hindered the establishment of a clear relationship between AATD and cardiovascular disease [16].

A series of clinical studies and animal models have attempted to explore the role of AAT and its deficiency in the progression of atherosclerosis and, more broadly, on the cardiovascular system. All these studies seem to converge on the hypothesis that AAT is somehow crucial in maintaining the stability and integrity of the vascular wall, but how this translates into clinical terms remains to be clarified [16]. In 2001, it was first reported that AAT levels may be higher in patients with coronary atherosclerosis [46]. Accordingly, the association between increased AAT levels and the occurrence of myocardial infarction in individuals who did not have any cardiovascular risk factors was later documented [47]. Considering its role as an acute-phase protein, it was hypothesized that these peaks of serum AAT may be related to the cytoprotective role that AAT exerts on vascular endothelium [48], thus even suggesting the possibility that AAT replacement therapies could potentially mitigate the damage caused by the ischemia and reperfusion phases in myocardial tissues. In 2016, this hypothesis was validated in murine models of acute myocardial injury, demonstrating that AAT may have the ability to reduce the size of the ischemic area and preserve systolic function, independent of the effects caused by neutrophilic elastase [49]. Accordingly, the administration of AAT to patients with ST-segment elevated myocardial infarction (STEMI), in combination with standard therapeutic protocols, was found to be well-tolerated and effective in reducing the inflammatory processes associated with the cardiac event [22]. In keeping with this, data from a large Swiss cohort suggested a possible association between AATD and the prevalence of subclinical atherosclerosis and hypertension [19]. In contrast, later studies failed in confirming these results on hypertension [50,51,52], and an even inverse association was documented in two large Danish cohorts [17,18]. In one of these [18], it was documented that there was a lower prevalence of hypertension in AATD subjects, with values that were 5 mmHg lower for systolic and 2 mmHg lower for diastolic blood pressure compared to the general population. Within the context of their cohort study, the same authors also attempted to provide an initial meta-analytical information regarding the risk of ischemic heart disease in AATD. Given that their study primarily focused on the analysis of their epidemiological data, they did not conduct any subgroup or sensitivity analyses, nor did they implement any meta-regression models, including only a quarter of the patients considered in our meta-analysis. Nevertheless, it is noteworthy that their findings align with our conclusions.

Based on the information provided so far, it appears challenging to provide a full pathophysiological explanation and univocal interpretation for our results, which are however substantiated by large and high-quality epidemiological data, as our pooled analysis encompassed over 24,000 individuals with AATD and over half a million controls without. Additionally, while meta-regressions indicate that our results are not affected by AATD genotypes, it is worth noting in our analyses a larger effect size associated with the increase in patients requiring augmentation therapy. Overall, this may suggest the possibility of an even lower cardiovascular risk in more severe forms of AATD. The fact that the prevalence of COPD, which is itself an independent risk factor for cardiovascular disease [24,25], did not influence our results in meta-regressions suggests that AATD may activate some independent and still poorly understood pathophysiological mechanism.

Dahl et al. [17], who were among the first to document a decreased cardiovascular risk in the large Copenhagen City Heart Study cohort, proposed the hypothesis that alterations in the proportion of elastin in large elastic arteries could be attributed to the unrestricted elastase activity within the vessel wall of AATD patients, and that this may affect their distensibility, potentially leading to a decrease in blood pressure. Nearly two decades later, the same research group, while confirming their previous findings of a reduced cardiovascular risk in a larger Danish cohort, continued to propose that alterations in the elastic properties of the tunica media could substantially explain their results [18]. In support of this hypothesis, a study in 1997 documented lower stiffness in the abdominal aorta of AATD patients [53], ultimately leading to weakened wall integrity [54]. However, another study challenged this finding by reporting increased arterial stiffness in AATD patients, as indicated by higher values of aortic pulse-wave velocity [52]. Echocardiography further confirmed a reduced distensibility and abnormal strain of the aortic wall in AATD patients, along with larger ascending aorta diameters [55]. Thus, it started to emerge the hypothesis that local deficiency of AAT in large elastic arteries may contribute to the proteolytic damage of the arterial wall, thereby facilitating arterial enlargement and dissection [56]. Notably, various cardiovascular complications, such as aortic [57] and intracerebral aneurysms [58], descending aorta dissection [55], coronary and cervical artery dissection [59], and left ventricular pseudoaneurysm [54] have been observed in AATD patients.

Overall, the available evidence seems to suggest that the imbalance between elastase and protease in large elastic arteries of individuals with AATD may compromise the integrity of the arterial wall, leading to reduced elasticity and progressive enlargement [18]. This, in turn, may potentially elevate the risk of aneurysm and dissection rather than that of ischemic events [16]. However, we are currently unable to fully explain this phenomenon and we cannot even rule out the hypothesis that this ischemic risk reduction may somehow be related to the increased cardiopulmonary monitoring that patients diagnosed with AATD, especially if severe, undergo. Moreover, we believe that our “mechanical” explanation alone may not fully account for our findings, as it overlooks the role of AAT as an acute-phase protein and its immuno-modulatory and anti-inflammatory activities, including its direct interaction with interleukin 8 and leukotriene B4 as well as its down-regulation of tumor necrosis factor-α pathways [60]. Along with the reduced blood pressure [18], another aspect to consider when interpreting our findings is the concurrent impact of other traditional cardiovascular risk factors. In this regard, it has been demonstrated that AATD patients may exhibit lower serum concentrations of triglycerides and low-density lipoprotein cholesterol compared to controls, indicating impaired hepatic lipid secretion [18,20]. This additional factor may further contribute to the reduction in ischemic risk.

Collectively, irrespective of speculations on this matter, our meta-analysis results emphasize the urgent need for clinical and preclinical studies to better understand the whole spectrum of comorbidities associated with this often underrecognized and disabling condition, thus facilitating the identification of more personalized pharmacological and rehabilitation strategies [61,62,63,64].

### Limitations

It is important to recognize some limitations in our study. Firstly, as meta-analyses depend on pooled data, the observation of a high degree of heterogeneity is the greatest problem. As a matter of fact, AATD is a condition with a highly variable clinical spectrum, influenced by genotype and environmental factors, especially cigarette smoking. Considering that AAT levels are lower by 84% in PiZZ, 49% in PiSZ, 17% in PiMZ, and only 7% in PiSS individuals [6], the substantial variability in AATD severity among studies constitutes a significant contributor to the observed heterogeneity. Moreover, it is noteworthy that more than 75% of AATD cases in this meta-analysis were PiMZ, thus suggesting that our results may not be applicable to all AATD genotypes. Additionally, if we consider that COPD acts by itself as an independent cardiovascular risk factor, another source of heterogeneity comes from the highly variable rate of AATD subjects and non-AATD controls diagnosed with COPD across studies. However, one of the greatest sources of heterogeneity in our meta-analysis arises from the different prevalence of hypertension, diabetes, and dyslipidemia among the different populations involved, thus significantly influencing the baseline cardiovascular risk of our study participants. This significant heterogeneity, however, offered us the opportunity to use appropriate statistical models that allowed us to refine our findings and provide new information that individual studies alone could not provide. Firstly, by using the random effects method, we were able to take into account both within-study and between-study variance. Moreover, we implemented a number of meta-regression models that explored whether the difference between cases and controls in terms of ischemic heart disease could depend on key clinical and demographic variables. However, the limited number of studies coupled with the inability to retrieve important clinical or demographic predictors across all included studies restricted our ability to perform meta-regressions on a comprehensive range of potential confounders, including some relevant cardiovascular risk factors (e.g., dyslipidemia and smoking). A similar limitation due to the scarcity of studies was also encountered in the assessment of publication bias. Therefore, we must stress the importance of exercising great caution in interpreting the results of our meta-analysis.

Additionally, it should be considered that the majority of participants included in our meta-analysis are from a few large population studies, with Nakanishi et al. [31] accounting for ≈80% of the overall AATD population. However, it is noteworthy that conducting additional analyses by excluding one study at a time did not yield relevant changes in the overall results. Thus, even after excluding this largest study, our findings on the risk of ischemic heart disease in AATD were substantially confirmed. Overall, while recommending great caution in the interpretation of our results, we are quite confident that this can (at least in part) mitigate the aforementioned limitation.

Another significant limitation pertains to the design of the studies included in our pooled analyses, as only half were prospectively designed to specifically investigate cardiovascular comorbidities in this clinical context. Nevertheless, it is noteworthy that our results remained consistent when analyzing these prospective studies separately, with an even greater effect size observed.

## 5. Conclusions

In summary, individuals with AATD, especially those without a low concurrent metabolic risk, may exhibit a reduced risk of ischemic heart disease and myocardial infarction, even in the presence of mild deficiency of the serine protease inhibitor. While caution is warranted due to the observational nature of the data and the relevant limitations of our meta-analysis, these findings align with a substantial body of epidemiological evidence indicating that the frequency of ischemic cardiac complications is lower in this patient population, whereas that of dissection and aneurysm may be higher. Consequently, additional studies are needed to validate these findings and comprehensively characterize the range of comorbidities in this clinical context. This will facilitate effective disease management, identification of pharmacological and rehabilitation strategies, and the implementation of primary and secondary prevention protocols.

## Figures and Tables

**Table 1:** Demographic and clinical data of individuals with alpha-1 antitrypsin deficiency (AATD) and controls in included studies.

Study	Subjects (n)	Males n (%)	Age (Years)	BMI (kg/m^2^)	Diabetes n (%)	Hypertension n (%)	Dyslipidemia n (%)	Smokers n (%)	Ex-smokers n (%)	COPD n (%)
Dahl 2003 [17]	546 AATD	-	-	-	-	-	-	-	-	-
	9064 Controls	-	-	-	-	-	-	-	-	-
Elzouki 1999 [29]	6 AATD	6 (100)	74.0	-	0	4 (66.7)	-	-	-	-
	74 Controls	74 (100)	72.0	-	4 (5.4)	29 (39.2)	-	-	-	-
Fähndrich 2017 * [27]	139 AATD	77 (55.4)	59.8	24.6	5 (3.6)	57 (41.0)	-	-	-	139 (100)
	2506 Controls	1495 (59.7)	65.3	27.1	356 (14.2)	1428 (57.0)	-	-	-	2506 (100)
Greulich 2017 [30]	590 AATD	298 (50.5)	61.0	-	161 (27.3)	374 (63.4)	44 (31.6)	-	-	189 (32.0)
	5900 Controls	2980 (50.5)	61.1	-	1558 (26.4)	3863 (65.5)	987 (39.4)	-	-	5900 (100)
Nakanishi 2020 [31]	19,003 AATD	8650 (45.5)	57.9	27.3	-	-	-	1801 (9.5)	6607 (34.8)	789 (4.1)
	398,424 Controls	182,344 (45.8)	58.0	27.4	-	-	-	41,735 (10.5)	21,525 (53.3)	15,502 (3.9)
Tanash 2020 [26]	1545 AATD	768 (50.0)	47.0	-	30 (1.9)	63 (4.1)	24 (1.5)	130 (8.4)	713 (46.1)	711 (46.0)
	5883 Controls	2977 (50.0)	45.0	-	95 (1.6)	184 (3.1)	52 (0.9)	1528 (26.0)	3115 (52.9)	219 (3.7)
Winther 2022 ** [18]	392 AATD	173 (44.1)	55.8	26.2	-	86 (21.9) §	-	43 (11.0)	157 (40.0)	125 (31.9)
	91,148 Controls	40,942 (44.9)	57.8	26.2	-	17,898 (19.6) §	-	15,861 (18.1)	35,533 (39.0)	5137 (5.6)
Winther 2023 [28]	2209 AATD	1154 (52.2)	44.8	-	-	408 (18.5)				1167 (52.8)
	21,869 Controls	11426 (52.2)	44.7	-	-	3900 (17.8)				1500 (6.8)

BMI: body mass index; COPD: chronic obstructive pulmonary disease. Continuous data are reported as mean values, unless otherwise indicated. The minus sign indicates that the information has not been specifically provided and/or cannot be inferred from the text of the article. * Age and BMI are reported as median values. ** Data on cardiovascular outcomes are available for 390 cases and 90,934 controls. § Participants on antihypertensive therapy.

**Table 2:** Genotypes and augmentation therapy in individuals with alpha-1 antitrypsin deficiency (AATD) in included studies.

Study	Subjects (n)	PiZZ n (%)	PiSZ n (%)	PiMZ n (%)	PiSS n (%)	AT n (%)
Dahl 2003 [17]	546 AATD	8 (1.5)	12 (2.2)	514 (94.1)	12 (2.2)	0
Elzouki 1999 [29]	6 AATD	0	0	6 (100)	0	0
Fähndrich 2017 * [27]	139 AATD	106 (76.3)	2 (1.4)	-	-	110 (79.1)
Greulich 2017 [30]	590 AATD	-	-	-	-	-
Nakanishi 2020 [31]	19,003 AATD	140 (0.7)	867 (4.6)	16,983 (89.4)	1013 (5.3)	0
Tanash 2020 [26]	1545 AATD	1545 (100)	0	0	0	0
Winther 2022 ** [18]	392 AATD	185 (47.2)	207 (52.8)	0	0	0
Winther 2023 *** [28]	2209 AATD	876 (88.1)	118 (11.9)	0	0	0

PiZZ: protease inhibitor ZZ; PiSZ: protease inhibitor SZ; PiMZ: protease inhibitor MZ; PiSS: protease inhibitor SS; AT: augmentation therapy. The minus sign indicates that the information has not been specifically provided and/or cannot be inferred from the text of the article. * A total of 31 AATD subjects with unknown genotype. ** Data on cardiovascular outcomes are available for 390 cases and 90,934 controls. *** Data on genotype are available for 994 individuals with AATD.

**Table 3:** Sensitivity and subgroup analyses for the risk of ischemic heart disease in individuals with alpha-1 antitrypsin deficiency (AATD) compared with non-AATD controls. Panel A: “better quality” studies (Newcastle-Ottawa Scale ≥ 6); Panel B: exclusion of studies identifying AATD on the basis of serum AAT levels or medical history; Panel C: exclusion of studies where the participants could potentially be a subset of those enrolled in other studies conducted within the same region or country but with a larger sample size; Panel D: prospective studies; Panel E: retrospective studies. N: number; OR: odds ratio; 95%CI: 95% confidence interval.

	*N. of Studies*	*N. of Datasets*	*N. of Patients*	*Effect Size*
**SENSITIVITY ANALYSES**	**A. “Better quality” studies**			
	6	6	23,283 AATD 532,068 controls	OR: 0.826 (95%CI: 0.756–0.903); ***p* < 0.001** I^2^ = 40.6%; *p* = 0.135
	**B. Exclusion of studies without AATD genotyping**			
	6	6	22,080 AATD 510,279 controls	OR: 0.807 (95%CI: 0.723–0.901); ***p* < 0.001** I^2^ = 46.2%; *p* = 0.098
	**C. Exclusion of potentially duplicate populations**			
	7	7	24,289 AATD 532,148 controls	OR: 0.825 (95%CI: 0.749–0.908); ***p* < 0.001** I^2^ = 43.6%; *p* = 0.100
**SUBGROUP ANALYSES**	**D. Prospective**			
	4	4	4483 AATD 36,158 controls	OR: 0.641 (95%CI: 0.421, 0.977); ***p* = 0.039** I^2^ = 88.4%; *p* < 0.0001
	**E. Retrospective**			
	4	4	20,145 AATD 413,462 controls	OR: 0.831 (95%CI: 0.799, 0.865); ***p* < 0.001** I^2^ = 0%; *p* = 0.430

## Data Availability

Data sharing is not applicable to this article as no new data were created or analyzed in this study.

## Supplementary Materials

The following supporting information can be downloaded at: https://www.mdpi.com/article/10.3390/jcm12206490/s1, Table S1: Literature search (4 October 2023); Table S2: Functional parameters in individuals with alpha-1 antitrypsin deficiency (AATD) and controls in included studies; Table S3: Definition of ischemic heart disease in individuals with alpha-1 antitrypsin deficiency (AATD) and controls in included studies; Table S4: Assessment of quality of studies (Newcastle-Ottawa scale); Table S5: Assessment of publication bias for studies evaluating ischemic heart disease in individuals with alpha-1 antitrypsin deficiency (AATD) and controls; Table S6: Meta-regression analyses. Impact of alpha-1 antitrypsin genotypes and differences (Δ) in key clinical and demographic variables on the risk of ischemic heart disease in individuals with alpha-1 antitrypsin deficiency (AATD) compared with controls.
