# Supplementary material for: Cardiovascular Risk Associated with Alpha-1 Antitrypsin Deficiency (AATD) Genotypes: A Meta-Analysis with Meta-Regressions
Source: J Clin Med. 2023 Oct 12;12(20):6490. doi: 10.3390/jcm12206490 (PMC10607733; doi:10.3390/jcm12206490)
Supplement: Supplementary file 1 [file jcm-12-06490-s001.zip › jcm-2613212-supplementary.pdf]

Supplementary Materials

Cardiovascular Risk Associated with Alpha-1 Antitrypsin Deficiency (AATD) Genotypes: A Meta-Analysis with Meta-Regressions

Pasquale Ambrosino 1,*,† , Giuseppina Marcuccio 2,†, Carmen Lombardi 2, Silvestro Ennio D'Anna 2, Stefano Sanduzzi Zamparelli 3 , Costantino Mancusi 4 , Giorgio Alfredo Spedicato 5 , Andrea Motta 6,‡ and Mauro Maniscalco 2,3,*,‡

[1] Istituti Clinici Scientifici Maugeri IRCCS, Directorate of Telese Terme Institute, 82037 Telese Terme, Italy
[2] Istituti Clinici Scientifici Maugeri IRCCS, Pulmonary Rehabilitation Unit of Telese Terme Institute, 82037 Telese Terme, Italy; giuseppina.marcuccio@icsmaugeri.it (G.M.); carmen.lombardi@icsmaugeri.it (C.L.); silvestro.danna@icsmaugeri.it (S.E.D.)
[3] Department of Clinical Medicine and Surgery, Federico II University, 80131 Naples, Italy; stefanosanduzzi@gmail.com
[4] Department of Advanced Biomedical Science, Federico II University, 80131 Naples, Italy; costantino.mancusi@unina.it
[5] Department of Statistics and Quantitative Methods, Milano-Bicocca University, 20126 Milan, Italy; spedicato_giorgio@yahoo.it
[6] Institute of Biomolecular Chemistry, National Research Council, 80078 Pozzuoli, Italy; andrea.motta@icb.cnr.it
* Correspondence: pasquale.ambrosino@icsmaugeri.it (P.A.); mauro.maniscalco@icsmaugeri.it (M.M.)
† These authors contributed equally to this work.
‡ These authors also contributed equally to this work.

Table of contents:

**Table S1**	**Literature search (October 4, 2023).**
**Table S2**	**Functional parameters in individuals with alpha-1 antitrypsin deficiency (AATD) and controls in included studies.**
**Table S3**	**Definition of ischemic heart disease in individuals with alpha-1 antitrypsin deficiency (AATD) and controls in included studies.**
**Table S4**	**Assessment of quality of studies (Newcastle-Ottawa scale).**
**Table S5**	**Assessment of publication bias for studies evaluating ischemic heart disease in individuals with alpha-1 antitrypsin deficiency (AATD) and controls.**
**Table S6**	**Meta-regression analyses. Impact of alpha-1 antitrypsin genotypes and differences (Δ) in key clinical and demographic variables on the risk of ischemic heart disease in individuals with alpha-1 antitrypsin deficiency (AATD) compared with controls.**

Table S1. Literature search (October 4, 2023).

Search terms in PubMed	Number of results
(α1 antitrypsin deficiency OR α1-antitrypsin deficiency OR alpha-1 antitrypsin deficiency OR alpha-1-antitrypsin deficiency)	5,670
(α1 antitrypsin deficiency OR α1-antitrypsin deficiency OR alpha-1 antitrypsin deficiency OR alpha-1-antitrypsin deficiency) and (myocardial)	24
(α1 antitrypsin deficiency OR α1-antitrypsin deficiency OR alpha-1 antitrypsin deficiency OR alpha-1-antitrypsin deficiency) and (myocardial OR infarction)	26
(α1 antitrypsin deficiency OR α1-antitrypsin deficiency OR alpha-1 antitrypsin deficiency OR alpha-1-antitrypsin deficiency) and (myocardial OR infarction OR angina)	27
(α1 antitrypsin deficiency OR α1-antitrypsin deficiency OR alpha-1 antitrypsin deficiency OR alpha-1-antitrypsin deficiency) and (myocardial OR infarction OR angina OR cardiac)	290
(α1 antitrypsin deficiency OR α1-antitrypsin deficiency OR alpha-1 antitrypsin deficiency OR alpha-1-antitrypsin deficiency) and (myocardial OR infarction OR angina OR cardiac OR cardiovascular)	413
(α1 antitrypsin deficiency OR α1-antitrypsin deficiency OR alpha-1 antitrypsin deficiency OR alpha-1-antitrypsin deficiency) and (myocardial OR infarction OR angina OR cardiac OR cardiovascular OR ischemic)	438
(α1 antitrypsin deficiency OR α1-antitrypsin deficiency OR alpha-1 antitrypsin deficiency OR alpha-1-antitrypsin deficiency) and (myocardial OR infarction OR angina OR cardiac OR cardiovascular OR ischemic OR ischemia)	**440**
**Additional records identified through other sources (Scopus, Web of Science, EMBASE)**	**1,591**
**TOTAL**	**2,031**

Table S2. Functional parameters in individuals with alpha-1 antitrypsin deficiency (AATD) and controls in included studies.

Study	Subjects (n)	FEV_1 (L)	FEV_1 (% predicted)	FVC (L)	FVC (% predicted)	FEV_1/FVC	6MWD (m)
Dahl 2003	546 AATD	-	-	-	-	-	-
	9064 controls	-	-	-	-	-	-
Elzouki 1999	6 AATD	-	-	-	-	-	-
	74 controls	-	-	-	-	-	-
Fähndrich 2017 *	139 AATD	1.6	50.1	-	78.0	49.1	437.3
	2506 controls	1.7	56.6	-	78.6	55.0	418.3
Greulich 2017	590 AATD	-	-	-	-	-	-
	5900 controls	-	-	-	-	-	-
Nakanishi 2020	19003 AATD	2.8	94.0	-	-	77.0	-
	398424 controls	2.8	94.0	-	-	77.0	-
Tanash 2020	1545 AATD	-	-	-	-	-	-
	5883 controls	-	-	-	-	-	-
Winther 2022 **	392 AATD	-	-	-	-	68.8	-
	91148 controls	-	-	-	-	77.4	-
Winther 2023	2209 AATD	-	-	-	-	-	-
	21869 controls	-	-	-	-	-	-

FEV_1: forced expiratory volume in 1 s; FVC: forced vital capacity; 6MWD: 6-minute walking distance. Continuous data are reported as mean values, unless otherwise indicated. The minus sign indicates that the information has not been specifically provided and/or cannot be inferred from the text of the article.

* Spirometry parameters and 6MWD are reported as median values.
** Data on cardiovascular outcomes are from 390 patients and 90,934 controls.

Table S3. Definition of ischemic heart disease in individuals with alpha-1 antitrypsin deficiency (AATD) and controls in included studies.

Study	ID codes	Outcome description
Dahl 2003<br>*Copenhagen City Heart Study*	ICD 8th Edition: 410-414	• Acute myocardial infarction<br>• Other acute and subacute forms of ischaemic heart disease<br>• Chronic ischaemic heart disease<br>• Angina pectoris<br>• Asymptomatic ischaemic heart disease
Dahl 2003<br>*Copenhagen University Hospital*	-	• Acute myocardial infarction<br>• Severe stenosis on coronary angiography<br>• Positive result on exercise electrocardiography
Elzouki 1999	-	• Coronary heart disease requiring medication
Fähndrich 2017	-	• -
Greulich 2017	ICD 10th Edition: I25	• Chronic ischemic heart disease
Nakanishi 2020	Phecodes: 411, 411.1, 411.2, 411.3, 411.4, 411.41, 411.8, 411.9	• Angina pectoris<br>• Coronary atherosclerosis<br>• Other chronic ischemic heart disease, unspecified<br>• Myocardial infarction<br>• Unstable angina (intermediate coronary syndrome)<br>• Other acute and subacute forms of ischemic heart disease<br>• Aneurysm and dissection of heart
Tanash 2020	ICD 10th Edition: I20-I25	• Angina pectoris<br>• Acute myocardial infarction<br>• Subsequent myocardial infarction<br>• Certain current complications following acute myocardial infarction<br>• Other acute ischaemic heart diseases<br>• Chronic ischaemic heart disease
Winther 2022	ICD 10th Edition: I20-I25	• Angina pectoris<br>• Acute myocardial infarction<br>• Subsequent myocardial infarction<br>• Certain current complications following acute myocardial infarction<br>• Other acute ischaemic heart diseases<br>• Chronic ischaemic heart disease
Winther 2023	ICD 10th Edition: I21-I22	• Acute myocardial infarction

ICD: International Classification of Diseases.

Table S4. Assessment of quality of studies (Newcastle-Ottawa scale).

Cohort studies	Representativeness of the exposed cohort	Selection of the non-exposed cohort	Ascertainment of exposure	Demonstration that outcome of interest was not present at start of study	Comparability of cohorts on the basis of the design or analysis*	Assessment of outcome	Was follow-up long enough for outcomes to occur	Adequacy of follow up of cohorts	Quality
Elzouki 1999	✶	✶	✶		✶✶	-	-	-	5
Fähndrich 2017	✶	✶	-	-	✶✶	-	-	-	4
Greulich 2017	✶	✶	✶	-	✶✶	✶	-	-	6
Nakanishi 2020	✶	✶	✶		✶✶	✶	-	-	6
Tanash 2020	✶	✶	✶	-	✶✶	✶	✶	✶	8
Winther 2022	✶	✶	✶	-	✶✶	✶	-	-	6
Winther 2023	✶	✶	✶	-	✶✶	✶	-	-	6

Case-control studies	Adequate case definition	Representativeness of the cases	Selection of controls	Definition of controls	Comparability of cases and controls on the basis of the design or analysis*	Ascertainment of exposure	Same method of ascertainment for cases and controls	Non-response rate	Quality
Dahl 2003	-	✶	✶	✶	✶✶	✶	✶	✶	8

***A maximum of 2 stars can be allotted in this category: ✶ if enrolling controls matched to cases for age and sex; ✶✶ if enrolling controls matched to cases for any additional factor. N/A: not applicable.**

Table S5. Assessment of publication bias for studies evaluating ischemic heart disease in individuals with alpha-1 antitrypsin deficiency (AATD) and controls.

**Egger's regression intercept**	Intercept	-1.19022
	Standard error	0.91411
	95% lower limit	-3.42697
	95% upper limit	1.04652
	t-value	1.30205
	P value (2-tailed)	0.24065
**Begg and Mazumdar**	**Kendall's S statistic (P-Q)**	-8.00000
	**Kendall's tau without continuity correction**	
	tau	-0.28571
	Z-value for tau	0.98974
	P-value (2-tailed)	0.32230
	**Kendall's tau with continuity correction**	
	tau	-0.25000
	Z-value for tau	0.86603
	P-value (2-tailed)	0.38648

**Duvall and Tweedie's trim and fill**	**Studies trimmed/imputed**	**Point estimate**	**Lower limit**	**Upper limit**	**Q value**
Observed values	-	0.77851	0.66465	0.91188	29.57446
Adjusted values (on the left)	0	0.77851	0.66465	0.91188	29.57446
Adjusted values (on the right)	1	0.78236	0.66639	0.91852	31.59239

Table S6. Meta-regression analyses. Impact of alpha-1 antitrypsin genotypes and differences (Δ) in key clinical and demographic variables on the risk of ischemic heart disease in individuals with alpha-1 antitrypsin deficiency (AATD) compared with controls.

Independent variable	Main results	Proportion of total between-study variance explained by the model	Goodness of fit: Test that unexplained variance is zero
$\Delta_{\text{\% of males}}$	**Z-score: 3.40, P<0.001**	R^2 analog: 0.30	Q: 20.22, df: 5; P=0.001
Δ_{Age}	Z-score: 1.76, P=0.078	R^2 analog: 0.00	Q: 29.17, df: 5; P<0.001
Δ_{BMI}	N/A	N/A	N/A
$\Delta_{\text{\% of diabetes}}$	**Z-score: 4.25, P<0.001**	R^2 analog: 1.00	Q: 1.07, df: 2; P=0.584
$\Delta_{\text{\% of hypertension}}$	**Z-score: 2.31, P=0.021**	**R^2 analog: 0.24**	**Q: 19.56, df: 4; P<0.001**
$\Delta_{\text{\% of dyslipidemia}}$	N/A	N/A	N/A
$\Delta_{\text{\% of smokers}}$	N/A	N/A	N/A
$\Delta_{\text{\% of ex-smokers}}$	N/A	N/A	N/A
$\Delta_{\text{\% of COPD}}$	Z-score: 0.25, P=0.799	R^2 analog: 0.00	Q: 26.97, df: 4; P<0.001
Δ_{FEV1}	N/A	N/A	N/A
$\Delta_{\text{FEV1\%}}$	N/A	N/A	N/A
Δ_{FVC}	N/A	N/A	N/A
$\Delta_{\text{FVC\%}}$	N/A	N/A	N/A
$\Delta_{\text{FEV1/FVC}}$	N/A	N/A	N/A
Δ_{6MWD}	N/A	N/A	N/A
PiZZ	Z-score: -1.48, P=0.138	R^2 analog: 0.00	Q: 21.05, df: 4; P<0.001
PiSZ	Z-score: 1.27, P=0.205	R^2 analog: 0.00	Q: 26.39, df: 4; P<0.001
PiMZ	Z-score: 0.63, P=0.530	R^2 analog: 0.00	Q: 7.27, df: 3; P=0.064
PiSS	Z-score: 0.36, P=0.715	R^2 analog: 0.00	Q: 8.45, df: 3; P=0.038
Augmentation therapy	**Z-score: -4.21, P<0.001**	**R^2 analog: 0.75**	**Q: 10.80, df: 5; P=0.055**

BMI: body mass index; COPD: chronic obstructive pulmonary disease; FEV_1: forced expiratory volume in 1 s; FVC: forced vital capacity; 6MWD: 6-minute walking distance; PiZZ: protease inhibitor ZZ; PiSZ: protease inhibitor SZ; PiMZ: protease inhibitor MZ; PiSS: protease inhibitor SS; N/A: not assessed because of the limited number of studies reporting this co-variate.
